# miRNA-34c Overexpression Causes Dendritic Loss and Memory Decline

**DOI:** 10.3390/ijms19082323

**Published:** 2018-08-08

**Authors:** Yu-Chia Kao, I-Fang Wang, Kuen-Jer Tsai

**Affiliations:** 1 Institute of Clinical Medicine, College of Medicine, National Cheng Kung University, Tainan 704, Taiwan; yukanomail2006@yahoo.com.tw (Y.-C.K.); e_fung0207@hotmail.com (I.-F.W.); 2 Department of Pediatrics, E-Da Hospital, Kaohsiung 824, Taiwan; 3 Institute of Molecular Biology, Academia Sinica, Taipei 115, Taiwan; 4 Research Center of Clinical Medicine, National Cheng Kung University Hospital, College of Medicine, National Cheng Kung University, Tainan 704, Taiwan

**Keywords:** miR-34c, dendritic spine, Alzheimer’s disease

## Abstract

Microribonucleic acids (miRNAs) play a pivotal role in numerous aspects of the nervous system and are increasingly recognized as key regulators in neurodegenerative diseases. This study hypothesized that miR-34c, a miRNA expressed in mammalian hippocampi whose expression level can alter the hippocampal dendritic spine density, could induce memory impairment akin to that of patients with Alzheimer’s disease (AD) in mice. In this study, we showed that miR-34c overexpression in hippocampal neurons negatively regulated dendritic length and spine density. Hippocampal neurons transfected with miR-34c had shorter dendrites on average and fewer filopodia and spines than those not transfected with miR-34c (control mice). Because dendrites and synapses are key sites for signal transduction and fundamental structures for memory formation and storage, disrupted dendrites can contribute to AD. Therefore, we supposed that miR-34c, through its effects on dendritic spine density, influences synaptic plasticity and plays a key role in AD pathogenesis.

## 1. Introduction

Alzheimer’s disease (AD) is a neurodegenerative disorder characterized by memory loss and cognitive decline due to extracellular accumulation of beta-amyloid peptide and intracellular accumulation of tau; it is also a consequence of dysfunction and loss of synapses [1]. From a pathological perspective, although neurofibrillary tangles and extracellular amyloid accumulations—defined as neuritic plaques—are the main hallmarks of AD, synaptic loss is the best predictor of clinical symptoms of AD [1].

Microribonucleic acids (miRNAs) are small noncoding RNAs composed of 19–25 nucleotides, which mediate the post-transcriptional regulation of gene expression. These non-coding RNAs function by binding to the 3′ untranslated regions of target messenger RNAs (mRNAs) (within the RNA-induced silencing complex), silencing their target mRNAs, and downregulating protein translation [2]. Each miRNA contains seed sequences crucial for recognizing and binding to target mRNAs. Some miRNAs participate in key neuronal functions, such as cell signaling, transcription, and axonal guidance [3]. More than three miRNAs, including miR-124, miR-34, and miR-132, are key to hippocampal function [4,5]. miR-124 expression in the medial prefrontal cortex could partially rescue the behavioral deficits associated with frontotemporal dementia [6]. Zovoilis et al. found that miR-34c was upregulated in AD patients [7] Bhatnagar et al. showed that there was a strong correlation between the expression level of miR-34c and scores of mini-mental state examination [8]. Other studies have observed that miR-132 is downregulated in patients with mild cognitive impairment [9], and the miR-132 level is reduced in AD-affected brains [10]. Therefore, miR-124, miR-34, and miR-132 are regarded as memory miRNAs, which play a key role in neurodegenerative diseases such as AD, Parkinson’s disease, and Huntington’s disease [5].

miR-34 is a markedly conserved miRNA of the let-7 family, with an identical seed sequence of orthologues in flies, *Caenorhabditis elegans*, mice, and humans [11]. The miR-34 family consists of three members: miR-34a, miR-34b, and miR-34c. Of these, miR-34a and miR-34c have identical seed sequences, whereas the seed sequence of miR-34b is similar, but not identical. This suggests that miR-34a and miR-34c share similar mRNA targets, whereas those of miR-34b might be slightly different [12]. Although miR-34b and miR-34c are co-transcribed from the same chromosome, they differ in amount and may regulate different targets in a particular brain region [13]. In mice, miR-34a is ubiquitously expressed, with its highest levels appearing in the brain, whereas miR-34b and miR-34c are expressed mainly in the brain, lungs, testes, and ovaries [14].

The miR-34 family is essential for normal brain development [14]; miR-34 has been linked to neurogenesis, spine morphology, neurite outgrowth, neurodegeneration, and hippocampal memory formation [15]. In zebrafish, the repression of miR-34 led to developmental defects in the neuronal system with an enlargement of the hindbrain during early embryonic development [16]. Moreover, miR-34/449 controls mitotic spindle orientation during mammalian cortex development [17]. Furthermore, miR-34 has been implicated in brain disorders and aging; in *Drosophila*, loss of miR-34 decreased survival and accelerated brain aging and degeneration, whereas miR-34 upregulation extended survival and diminished neurodegeneration due to human pathogenic polyglutamine disease protein [11]. In addition to being present in neurons, miR-34 is expressed in glial cells [4].

Zovoilis et al. showed that miR-34c was elevated in the hippocampi of AD patients, aging mice, and APPPS1-21, a model of amyloid pathology linked to AD. The researchers demonstrated that miR-34c constrained memory consolidation, and miR-34c-mediated memory impairment was regulated, at least in part, by decreased hippocampal sirtuin-1 (SIRT1) levels [7]. Similarly, the expression of another miR-34 family member—miR-34a—was elevated in APPswe/PS∆E9 (AD mouse model) mice compared with control mice [18]. However, a contradictory correlation between miR-34 and cognitive function was also observed. A study that used Adeno-associated virus-delivered sponges in mice revealed that miR-34 inhibition impaired reference memory during the Morris water maze [4]. In *Drosophila*, loss of miR-34 accelerated brain aging and late-onset brain degeneration. miR-34 mutant flies were born with typical brains but showed dramatic vacuolization with age, whereas miR-34 upregulation extended their median lifespan and mitigated neurodegeneration [11]. Because AD is a neurodegenerative disorder associated with aging, we were unsure why loss of miR-34 accelerated brain aging in *Drosophila* while its overexpression caused memory impairment in mammals. Dickson et al. observed that miR-34a inhibited expression of endogenous tau, a crucial intraneuronal aggregate of AD [19]. In addition, Wu et al. found that overexpression of miR-34c downregulated tau expression in gastric cancer cells [20], although the researchers did not perform experiments to test cognitive function or analyze tau proteins in neural cells, the results were confusing in that the decreased tau expression associated with miR-34a and miR-34c overexpression contradicted AD pathogenesis. Therefore, miR-34 seems to play a mutual role in neurodegeneration; it can be protective or causative. For this reason, we wanted to study the role of miR-34c in memory function in mammals.

Dendritic spines are tiny protrusions along dendrites that constitute major postsynaptic sites for synaptic transmission [21]. These spines are highly dynamic structures that can undergo remodeling even in adults. Spine remodeling and new synapse formation, termed “plasticity”, underlie the basis for learning and memory [22]. Loss or alteration of these structures was described in patients with neurodegenerative disorders such as AD [23], and synaptic reduction is the feature most closely related with decline in memory and cognition [24]. In one study, the density of neocortical synapses revealed highly powerful correlations with cognitive alterations in AD—the lower the mental status scores, the greater was the loss of synapses [25]. In another study, only weak correlations were observed between psychometric indices and plaques and tangles [26]. Thus, AD is primarily considered to be a synaptopathy [27].

Based on previous reports regarding the importance of particular hippocampal miRNAs in memory function, we studied the influence of miR-34c on memory function and compared dendritic spines between hippocampal neurons overexpressing miR-34c and controls, given that dendritic spines are the fundamental elements of synapses that form the basis of learning and memory.

## 2. Results

### 2.1. Overexpression of miR-34c Caused Memory Impairment

**Figure 1:**
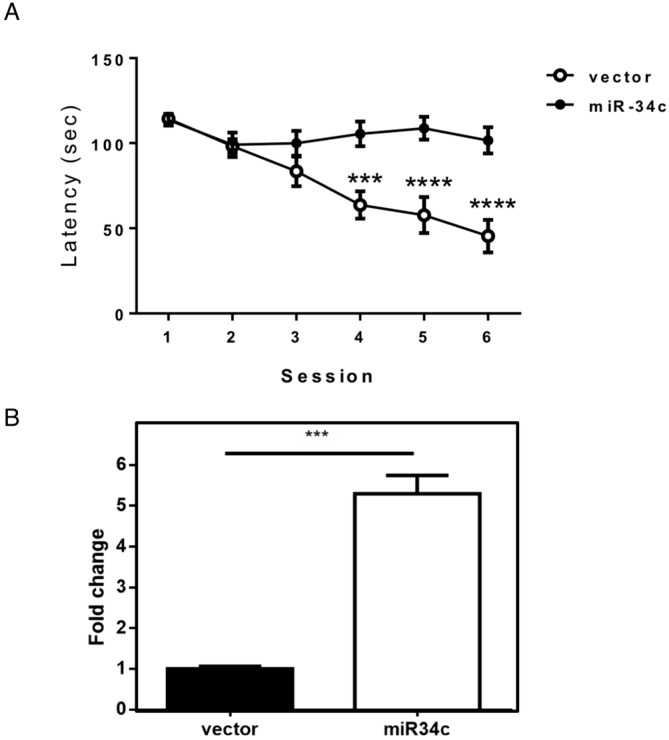
Overexpression of miR-34c caused memory impairment. (**A**) Escape latencies of mice overexpressing miR-34c and control vector (vec) mice (*n* = 16 in each group) in the Morris water maze. The asterisks represent the level of statistical significance calculated by a two-way analysis of variance (*** *p* < 0.001, **** *p* < 0.0001); (**B**) Hippocampal miR-34c expression levels were determined by quantitative PCR in mice transfected with miR-34c construct or mice transfected with the control vector and calculated using a two-tailed Student’s *t*-test (*** *p* < 0.001).

Using the Morris water maze task, we found that mice overexpressing miR-34c exhibited markedly increased latency compared with vector-transfected (control) mice (Figure 1A), indicating that the mice overexpressing miR-34c exhibited memory impairment. We used quantitative polymerase chain reaction (PCR) to measure the expression level of miR-34c in the hippocampi of miR-34c and control mice. The miR-34c expression level of the miR-34c-transfected mice was five times higher than that of the control mice (Figure 1B).

### 2.2. Expression Level of miR-34c in the Hippocampus

**Figure 2:**
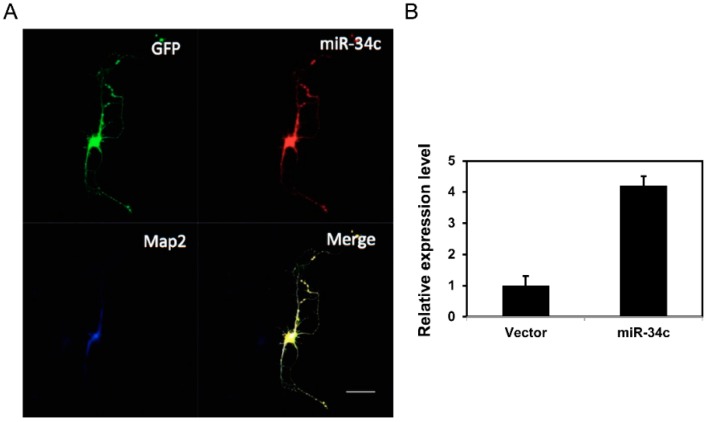
We observed that miR-34c was expressed in cultured hippocampal neurons. (**A**) Transfection of miR-34c was identified on DIV7 through immunofluorescence staining. Cells co-expressing green fluorescence (from GFP^+/−^ mice) and red fluorescence (from miR-34c-transfected cells) were selected, and neural dendrites were confirmed using the Map2 antibody. Scale bar = 50 μm; (**B**) The expression level of miR-34c in the cultured hippocampal cells transfected with the miR-34c construct or control vector were detected using RT-PCR and calculated using a two-tailed Student’s *t*-test.

The miR-34c construct was transfected into primary hippocampal cells to investigate the role of miR-34c in neuronal morphology. The transfection efficiency of miR-34c was identified on the seventh day of culture (DIV7) through immunofluorescence staining. Cells that co-expressed green fluorescence (from green fluorescent protein GFP^+/−^ mice) and red fluorescence (from miR-34c-transfected cells) were selected, and neural dendrites were confirmed using the microtubule-associated protein 2 (Map2) antibody, which stained the soma and dendrites, but not the axons (Figure 2A). The expression levels of miR-34c in the transfected and control cells were detected using reverse transcription PCR (RT-PCR). Of the hippocampal primary cells, the expression of miR-34c was nearly four times higher in the miR-34c-transfected cells than in the control cells (Figure 2B).

### 2.3. Overexpression of miR-34c Reduced Dendritic Length and Branch Numbers

**Figure 3:**
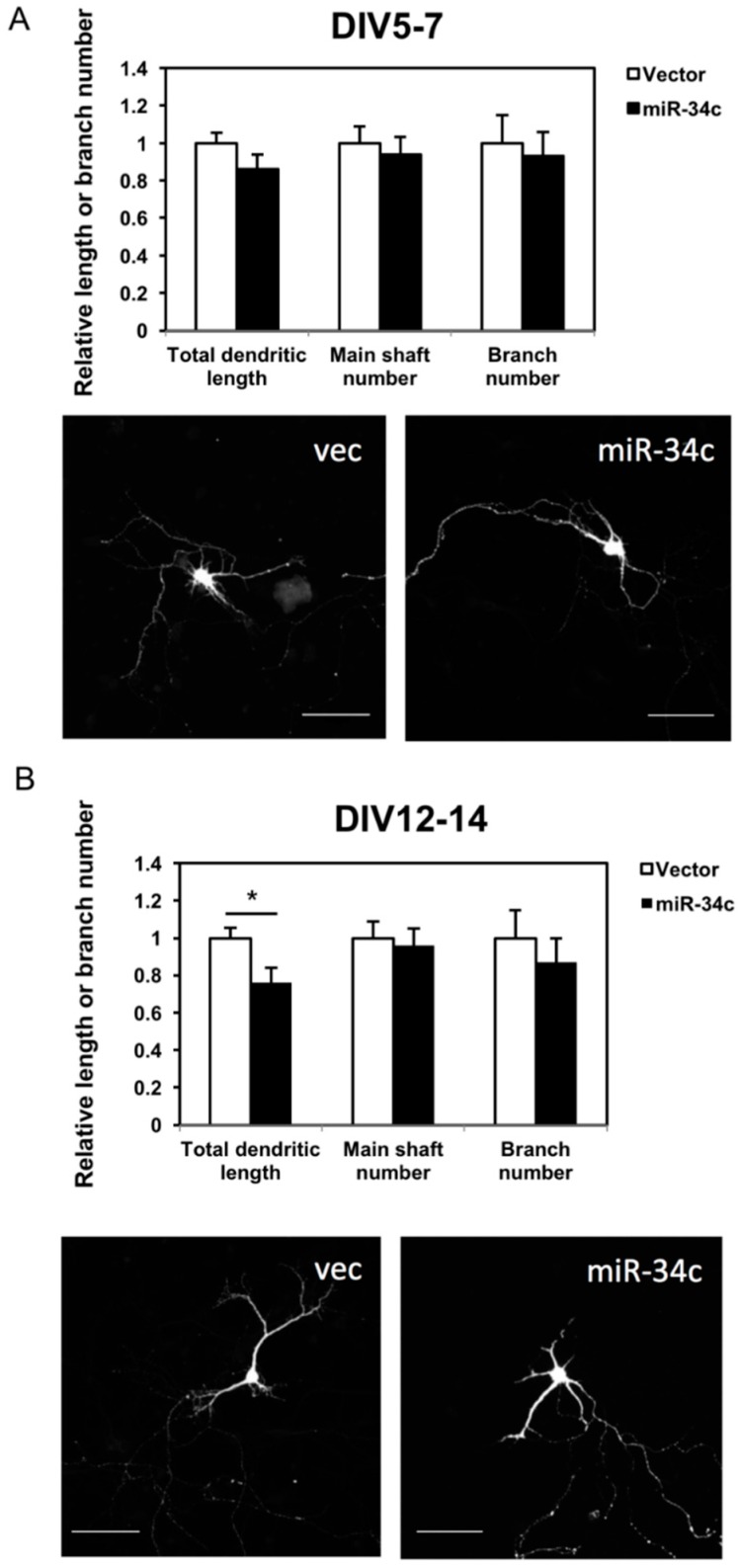
We observed that miR-34c affected the dendritic length of hippocampal neurons. Immunostaining with GFP, red fluorescent protein, and the Map2 antibody was performed on (**A**) DIV 7 (Scale bar = 100 μm) and (**B**) DIV 14. The total dendritic length, shaft number, and branch number of miR-34c hippocampal neurons and vector-transfected (vec) hippocampal neurons (*n* = 18 in each group) were counted under a fluorescence and confocal microscope (20× magnification) and calculated using a two-tailed Student’s *t*-test (* *p* < 0.05). Scale bar = 100 μm.

To investigate the effect of miR-34c on neuronal morphology, we measured the total dendritic length, dendritic main shaft, and dendritic branch numbers of the hippocampi two days after transfection. In the earlier stage—DIV 7—it appeared that miR-34c suppressed the outgrowth and branching of dendrites; however, this result was nonsignificant (Figure 3A). In the later stage—at DIV 14—the total dendritic length of neurons with miR-34c overexpression had a shorter dendritic length than did the neurons transfected with the control vector (Figure 3B). The numbers of dendritic shafts and branches were slightly decreased in the miR-34c-overexpression group; however, this result was nonsignificant. Therefore, miR-34c partially inhibited dendritic outgrowth but did not affect dendritic branching.

### 2.4. Effects of miR-34c Overexpression on Neurite Protrusion Density

**Figure 4:**
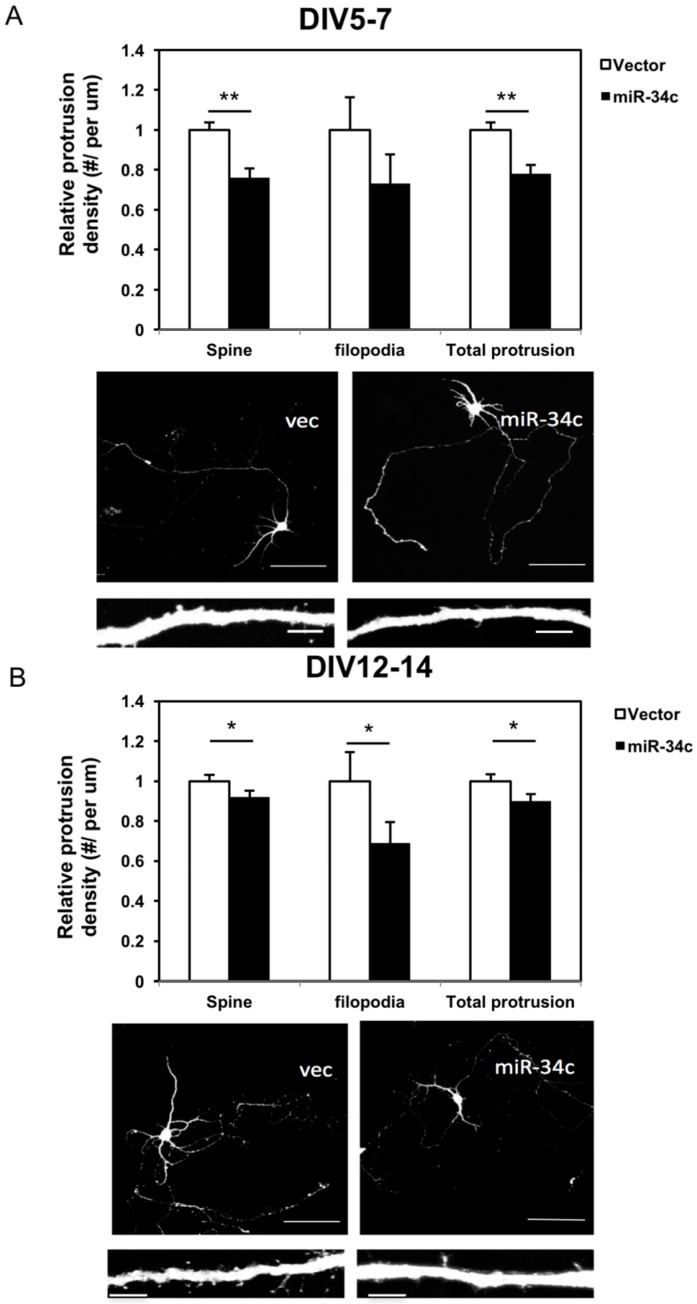
We observed that miR-34c lowered dendritic spine density and total protrusion density. Quantification of the protrusion density of neurites including mature spines, immature filopodia, and the summation of both using immunostaining on the (**A**) DIV 7 (Scale bar of upper panel = 100 μm; lower panel = 5 μm) and (**B**) DIV 14 in miR-34c and vector-transfected (vec) cells (*n* = 28 in each group) was conducted under 100× magnification and calculated using a two-tailed Student’s *t*-test (* *p* < 0.05, ** *p* < 0.01). Scale bar of upper panel = 100 μm; lower panel = 5 μm.

Protrusions of neurites develop at an early stage of neuronal development and later differentiate into mature spines. This process requires electrical signals from other neurons. The density of protrusions reflects not only the morphological state of the neurons, but also the physiological function. Therefore, we investigated the status of these protrusions by categorizing them into spines and filopodia, and measured the total protrusions, defined as the summation of spines and filopodia. Spines were defined as short extensions from the membranes of dendrite, with mushroom-like heads or extensions with an obscure neck. Filopodia were slender protrusions mostly twice longer as spines, and were identified as protrusions without an apparent shallow head; they were considered the immature form of protrusions. On DIV7, when the protrusions had not developed completely, neurons transfected with miR-34c had a significantly lower spine density and total number of protrusions (Figure 4A). On DIV14, the neurons transfected with miR-34c exhibited declines in spines and filopodia, as well as in total protrusions compared with the controls (Figure 4B).

### 2.5. Effects of miR-34c Overexpression on Protrusion Density in Various Dendritic Areas

**Figure 5:**
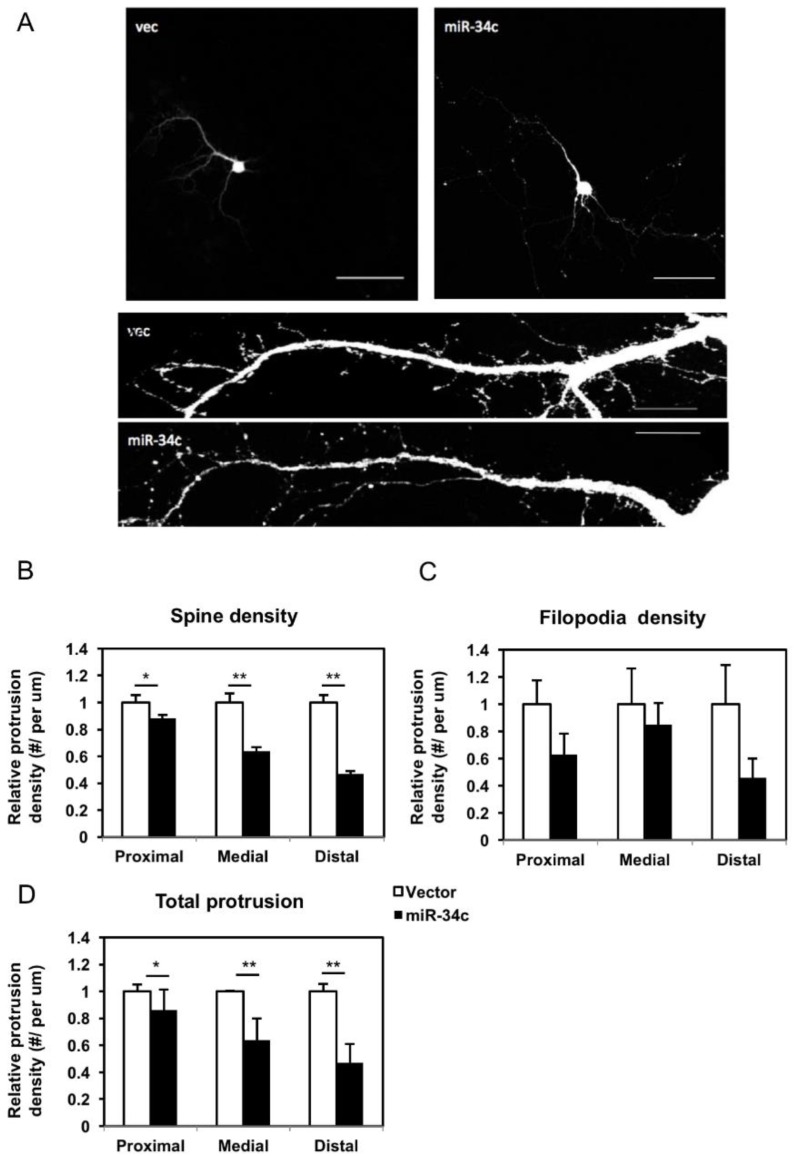
We observed that miR-34c exerted a negative effect on spine density along the dendrites. (**A**) Low- and high-power fields (20× in upper panels and 100× in lower panels) were used for counting. Scale bar of upper panel = 100 μm, lower panel = 20 μm. Quantification of the densities of (**B**) spines, (**C**) filopodia, and (**D**) total protrusions was conducted by dividing each dendrite into three equal segments of 40-μm in length, starting from the soma as the proximal segment (0–40 μm) and followed by the middle (40–80 μm) and distal (80–120 μm) segments, and calculated using a two-tailed Student’s *t*-test (* *p* < 0.05, ** *p* < 0.01).

To gain a better understanding of the effects of miR-34c on dendritic protrusion, each dendrite was divided into three equal segments of 40 μm for further analysis, starting from the soma as the proximal segment and followed by the middle and distal segments. The results revealed various density patterns in the spines and filopodia (Figure 5A). Spine density and total protrusions in all three segments of the miR-34c-transfected cells remained lower than those in the controls (Figure 5B,D). In addition, the reductions in spine density and total protrusions were proportional to their distance from the soma, meaning that in the miR-34c-overexpressing neurons, the farther the dendrites were from the soma, the lower was the number of spines. By contrast, no such change was observed in the filopodia along the dendrites (Figure 5C). Based on these findings, we assumed that miR-34c participated in the formation (turnover) of dendritic protrusions and may have acted as a negative regulator in the maturation of dendritic protrusions from filopodia into spines. While the spine density increased with the distance of dendrites from the soma in the control group, the miR-34c-transfected cells exhibited reduced spine density away from the soma.

## 3. Discussion

The present study showed that mice overexpressing miR-34c exhibited significant memory impairment and hippocampal dendritic spine loss. In our study, miR-34c partially inhibited dendritic outgrowth as the total dendritic length was shorter. In addition, miR-34c resulted in reduced numbers of dendritic spines and total protrusions at an early stage (DIV 7). Subsequently, the miR-34c-transfected cells exhibited loss of filopodia and spines; in these cells, the farther the dendrites were from the soma, the lower was the number of spines; however, no such change was observed among filopodia. A study on the acoustic and visual cortices of autopsied AD patients revealed a marked decrease in the number of dendritic spines and loss of distal spines. However, the branches of the apical part of the dendritic tree and basal arborizations appeared to be equally affected by spine depletion [28]. The hippocampus contains two types of dendrites on pyramidal cells: apical and basal dendrites. Apical dendrites can be further divided into distal and proximal parts. Pyramidal cells segregate their inputs by using proximal apical dendrites, which project radially to local pyramidal cells and interneurons, while distal apical dendrites form non-local synapses by receiving inputs from distant cortical and thalamic locations [29]. CA1 neurons receive inputs at the distal tuft from the entorhinal cortex through the perforant path and from the thalamic nucleus reuniens, whereas the basal and apical dendrites receive inputs from the CA3 through the Schaffer collaterals. Based on the finding of more prominent distal dendritic spinal loss in the miR-34c cells in our study, we assumed that miR-34c might disrupt the perforant path from the entorhinal cortex of the memory circuit in mammalian brains.

Dendritic abnormalities in AD are widespread and occur in early stages of the disease; such abnormalities can be divided into (1) dystrophic neurites, (2) dendritic complexity reduction, and (3) dendritic spine loss. Marked dendritic spine loss is the final major dendritic abnormality found in AD patients, and the cortex and hippocampus are the two areas most affected by spine loss [30,31]. In APPxPS1-KI (an AD mouse model), an alteration of spine morphology occurred before reduction of synapses and neuronal density. The researchers observed a reduction in spine length and enlargement its neck, giving the spines a more “stubby” appearance [32].

Filopodia are the smallest structures protruding from dendrites [33]. Filopodia-like protrusions are highly unstable and can form and be removed within hours, whereas larger mushroom-type spines are more likely to remain stable for months to years. In both cell culture and in vivo, filopodia can form, stabilize, and grow into larger spines; this suggests that they represent an early stage of spine formation [34]. Transitions in spine size and stability—from immature and unstable filopodia to mature and stable spines—accompany the maturation and strengthening of synaptic contacts on spines [33]. An in vivo imaging study on the rodent neocortex demonstrated that small spines were NMDAR-dominated and highly motile, whereas large spines were AMPAR-dominated and highly stable. These findings led to the hypothesis that small thin spines are learning spines, whereas larger mushroom spines are memory spines [35]. Changes in the shapes of spine heads (morphogenesis) were actin-dependent and likely regulated by synaptic stimulation in response to a variety of stimuli [36]. In the present study, in the miR-34c-transfected mice, the number of spines were decreased compared with the control mice at an early stage and the filopodia remained unaffected, whereas immature filopodia and mature spines were lost at a later stage; thus, we speculated that miR-34c might disrupt the maturation of spines at an earlier stage and eventually destroy all dendritic spines, thereby affecting learning and memory consolidation.

Studies by Zovoilis and Bhatnagar et al. showed that miR-34c was increased in AD patients’ brains and they pointed out one specific target—SIRT1—that contributed to memory decline [7,8]. Codocedo et al. later proved that SIRT1 overexpression was sufficient to change dendritic morphogenesis in the hippocampi and offer certain resistance against the cytotoxic damage induced by Aβ and avoid neuritic dystrophy [37]. Hence, the causal relationship between miR-34c and changes in dendritic spines in our study can be verified. Future research is required to investigate the pathogenic mechanism underlying the association between miR-34c and dendritic spine loss and to search for other possible miR-34c targets.

Structural changes in spines were driven by remodeling of the actin cytoskeleton [38]. Small Rho GTPases were deemed as central regulators of cell cytoarchitecture and played key roles in modulating cell migration, neurite outgrowth, survival, and synapse formation in neurons [39]. Cell division cycle 42 (Cdc42), ras-related C3 botulinum toxin substrate 1 (Rac1), and ras homolog family member A (RhoA) are the most frequently studied members of the small Rho GTPase family [40]. Rac and Rho are crucial to the maintenance of dendritic spines and branches in hippocampal pyramidal neurons [41] and appear to be critical in the formation and maintenance of memory [42]. Activation of Rac1 facilitated the formation of dendritic spines and increased spine head volume. By contrast, RhoA activation prevented spine formation and induced spine shortening [43]. As mentioned, miR-124, miR-34, and miR-132 are regarded as memory miRNAs. Both miR-124 and miR-132 have been reported to be associated with changes in dendritic spines through Rho GTPases, which underlie the stabilization of memory. In one study, miR-124 reduced the expression of Rho GTPs, thereby inhibiting axonal and dendritic branching via Rac1 and Cdc42 signaling, respectively [44]. Moreover, miR-132 activated Rac1 and promoted spine formation [45]. Thus far, no relationship between miR-34 and Rho GTPases in neurons has been reported. Since SIRT1 activators (RES) could modulates the dendritic arborization through the inhibition of Rho-associated protein kinase (ROCK) [37]—downstream effector of Rho GTPases—this served as indirect evidence for the association between miR-34c and Rho GTPases. Besides, one study investigated the roles of miR-34 and Rho GTPases in cancer cells; Huang et al. demonstrated that two key mechanisms involved in cancer metastasis—epithelial-to-mesenchymal transition and mesenchymal-to-amoeboid transition—were coupled through two miRNAs, namely miR-200 and miR-34, both of which inhibited RhoA and Rac1 [46]. This implies that Rho GTPase-dependent cytoskeletal changes might occur in the dendritic spines of miR-34-expressing hippocampal neurons, and thus underlie memory impairment.

Because postsynaptic density protein 95 (PSD-95) is the hallmark of a mature and stable glutamatergic synapse associated with memory consolidation [33], miR-34c might be associated with PSD-95. Bustos et al. designed an epigenetic editing strategy by using a zinc finger construct to control Dlg4/PSD-95 expression in the hippocampus and validate PSD-95 as a key player in plasticity and memory. PSD-95-6ZF-VP64 transduction could increase PSD-95 levels and recover learning and memory deficits in aged and AbPPswe/PS-1 (AD model) mice [47]. Whether synaptic changes in memory miRNAs are also related to PSD-95 requires further investigation.

## 4. Materials and Methods

### 4.1. Animals

C57BL/6JNarl (B6) mice were kept in a ventilated room under controlled conditions with a 12-h/12-h light/dark cycle and a maintained temperature of 22 ± 2 °C. The animals were given access to food and water ad libitum. This study was approved by the University Institutional Animal Care and Use Committee of National Cheng Kung University (NCKU) (Project code-106073, date of approval-23/12/2016). The experimental procedures for handling the mice were in accordance with the guidelines of the Institutional Animal Care and Use Committee of NCKU. Transgenic mice with GFP^+/−^ expression and B6 mice were provided by the national laboratory animal center.

### 4.2. Deoxyribonucleic Acid Constructions

To produce the miRNA constructs, the genomic deoxyribonucleic acid (DNA) from a B6-wild type mouse was used as the complementary template to amplify the precursor of miR-34c by PCR, with the forward primer: 5′-GCA GTG TAA TTA GCT GAT TGT AGT G-3′, and reverse primer: 5′-ATA TTA GGA AAC CAG CTG GT TTT AA-3′. After PCR amplification, the precursor miR-34c product was separated by electrophoresis and eluted from the agarose gel, ligated into a lentiviral construct-pFUGW, driven by a ubiquitin promoter, with a red fluorescent protein gene (dsRed) for co-expression.

### 4.3. Lentivirus Production

The lentivirus was produced by the RNAi core laboratory of Academia Sinica, Taipei, Taiwan with a 100× concentration.

### 4.4. Stereotaxic Hippocampal Injections

Male B6 mice were placed in the induction chambers. The oxygen flowmeter was adjusted to approximately 0.1–0.3 L/min. Isoflurane vaporizer was adjusted to 5% for anesthesia induction and 1.5–3.5% for maintenance. The anesthetized mice were then mounted in a stereotactic apparatus for intra-hippocampal injections. Concentrated lentivirus (1 × 10^7^ in 2 μL) was injected into the dentate gyrus of bilateral hippocampus (−2.0 mm anterior–posterior, 1.8 mm medial–lateral, and −2.3 mm dorsal–ventral relative to the bregma) using a Hamilton micro-syringe (Hamilton Company, Reno, Nevada). After each injection, the needle was left in situ for 8 min to prevent regurgitation of the virus during removal. Control groups were injected with the same volume of control lentivirus. A Morris Water Maze test was performed 30 days after the injection.

### 4.5. Morris Water Maze Test

miR-34c-expressing and control vector-infected male B6 mice, *n* = 16 in each group, were tested 30 days after hippocampal injections of viral particles. The swimming pool is divided into four quadrants and contains an escape platform placed beneath the water. The mice were trained with four consecutive trials per day for six days. When released, the mice swam around the pool in search of an exit and on subsequent trials the mice were able to locate the platform more rapidly. Each trial lasted until the mice found the platform or for a maximum of 2 min. The time to locate the platform was recorded and the average latency calculated from the values of four trials each day. The swimming speed and probe test were recorded on video for further analysis.

### 4.6. Primary Hippocampal Culture

The embryos from B6 wild-type pregnant female mice were used for hippocampal primary culture. Pregnant B6 wild-type mice were dissected at embryonic day 16.5, and the hippocampi were separated from embryonic brains. The hippocampal tissues were washed with 1× Hank’s balanced salt solution (HBSS) twice and digested with papain for about 10 min at 37 °C. Then, the aggregated tissues were washed with 1× HBSS twice, followed by resuspension in 1 ml Neurobasal medium with 1× B27 (Invitrogen, Thermo Fisher Scientific, Waltham, MA, USA), 1× Penicillin Streptomycin (P/S) antibiotics (Invitrogen), and 1× GlutaMax (Invitrogen). Cell viability was counted by an Invitrogen countess automatic cell counter. Cells were plated at 2 × 10^5^ cells/well on poly-d-lysine-coated coverslips in six-well plates and incubated at 37 °C/5% CO_2_. After 2 h, the medium was changed to a new Neurobasal medium to prevent contamination. Cells were cultured until DIV5 and DIV12 to perform miRNA transfection.

### 4.7. Transfection

Cells plated in six-well plates were transfected at DIV5 or DIV12. Thirty minutes before transfection, the medium was replaced with P/S antibiotics-free Neurobasal medium. The ubiquitin promoter-driven miR-34c plasmid, or vector only, were transfected into hippocampal primary culture with Lipofectamine 2000 (Invitrogen) for 2 h. The transfected cells were cultured for two days and analyzed by confocal microscopy for neuronal morphology or collected to extract RNA samples.

### 4.8. Immunofluorescence Staining

The transfected cells were washed twice with phosphate-buffered saline (PBS) and fixed with 4% paraformaldehyde (Electron Microscopy Sciences, Hatfield, UK). The cells were blocked for 1 h (blocking solution: 10% goat serum/PBS) at room temperature. The primary antibody was Map2 (Invitrogen) with 1% goat serum. All images were visualized using a Zeiss laser confocal microscope (LSM510, Zeiss, Oberkochen, Germany) and a fluorescence microscope (DeltaVision, GE Healthcare Life Sciences, London, UK), with a single optical section.

### 4.9. Image Analysis

Neural cells emitted green fluorescence because of the inherited GFP protein. All images were at the same resolution of 1024 pixels × 1024 pixels. Confocal z sectioning was used, and images were overlapped to have full signals. Each neuron was captured under the low-power field (20× objective, NA 1.4, respectively) for both soma and neurites. These low power images were used to measure the neuritic length and branch numbers, owing to the whole cell coverage with this larger visual field. Dendritic spines were focused on under high magnification (40× and 100× objective) in order to count spine numbers and observe spine morphology, specifically for measuring (1) total dendritic length, defined as the summation of all dendritic length in a neuron; (2) main dendritic shaft number, defined as the numbers of dendritic outgrowth from one soma; (3) dendritic branch number, defined as the total branch numbers from the primary dendrites; and (4) dendritic spine density, expressed as the average number of spines per μm of dendritic length. The total dendritic length and branch numbers were measured using the software NeuronJ (provided by Dr. Erik Meijering, Biomedical Imaging Group Rotterdam of Erasmus University Medical Center, Rotterdam, The Netherlands), whereas the numbers of different protrusions (spines, filopodia, and total protrusions) were counted using ImageJ (provided by Dr. Erik Meijering, Biomedical Imaging Group Rotterdam of Erasmus University Medical Center, Rotterdam, The Netherlands) in various areas divided by their distance from the soma (proximal: 0–40 μm, middle: 40–80 μm, distal: 80–120 μm).

### 4.10. Reverse Transcription PCR (RT-PCR)

The total RNA products extracted from the hippocampal primary culture or hippocampi in the brain were then reverse-transcribed to complementary DNA (cDNA) by SuperScript II RT (Invitrogen) following the Universal ProbeLibrary (UPL; Roche, Basel, Switzerland) protocol, and the expression levels of miR-34c were then detected by UPL probe #21, according to the manufacturer’s protocol. Primer sequences used were as followed, miR-34c UPL RT primer: GTT GGC TCT GGT GCA GGG TCC GAG GTA TTC GC ACCAGAGCCAACGCAATC, miR-34c forward primer: 5′-CGG CGA GGC AGT GTA GTT AGC T-3′, universal reverse primer: 5′-GTG CAG GGT CCG AGG T-3′.

### 4.11. Statistics

Data were analyzed using SPSS and the Morris water maze was analyzed using a two-way analysis of variance. Mean values were analyzed using a two-tailed Student’s *t*- test. All results are represented as mean ± standard error of the mean and were first examined using an *f*-test to identify the homogeneity of variance. A probability level of <0.05 was accepted as statistically significant and degrees of significance are presented as * *p* < 0.05, ** *p* < 0.01, *** *p* < 0.001, **** *p* < 0.0001.

## 5. Conclusions

We established miR-34c expression constructs and transfected miR-34c into primary hippocampal cells. The present study revealed that miR-34c overexpression resulted in memory decline, accompanied by a decrease in dendritic spine density. Our data suggested that miR-34c plays a pivotal role in cognitive decline and therapies targeting miR-34c may restore synaptic defects and shed further light on AD.
